# Phase‐Transited Lysozyme‐Driven Formation of Self‐Supported Co_3_O_4_@C Nanomeshes for Overall Water Splitting

**DOI:** 10.1002/advs.201900272

**Published:** 2019-04-05

**Authors:** Yuan Ha, Lingxia Shi, Ziliang Chen, Renbing Wu

**Affiliations:** ^1^ Department of Materials Science Fudan University Shanghai 200433 P. R. China

**Keywords:** bifunctional electrocatalysts, Co_3_O_4_@C nanomesh, lysozyme, overall water splitting

## Abstract

The development of highly efficient catalysts for both the hydrogen evolution reaction (HER) and oxygen evolution reaction (OER) is of paramount importance in water splitting. Herein, a phase‐transited lysozyme (PTL) is employed as the platform to synthesize nitrogen‐doped Co_3_O_4_@C nanomesh with rich oxygen vacancies supported on the nickel foam (N‐Co_3_O_4_@C@NF). This PTL‐driven N‐Co_3_O_4_@C@NF integrates the advantages of porous structure, high exposure of surface atoms, strong synergetic effect between the components and unique 3D electrode configuration, imparting exceptional activity in catalyzing both HER and OER. Remarkably, an alkaline electrolyzer assembled by N‐Co_3_O_4_@C@NF as both cathode and anode delivers a current density of 10 mA cm^−2^ at an ultralow cell voltage of 1.40 V, which is not only much lower than that of the commercially noble Pt/C and IrO_2_/C catalyst couple (≈1.61 V) but also a new record for the overall water splitting. The finding may open new possibilities for the design of bifunctional electrocatalysts for application in practical water electrolysis.

The ever‐increasing demands for sustainable energy have aroused a considerable interest to exploring new electrochemical energy conversion and storage technologies.^1^, ^2^, ^3^, ^4^, ^5^, ^6^, ^7^, ^8^ Electrochemical water splitting consisting of two half reactions, that is, the hydrogen evolution reaction (HER) at the cathode and the oxygen evolution reaction (OER) at the anode is regarded as one of the most promising ways to produce clean hydrogen fuel.^9^, ^10^, ^11^, ^12^, ^13^, ^14^, ^15^ However, both HER and OER are thermodynamically uphill and sluggish, and thus an efficient catalyst is required to lower the energy barrier and increase the reaction rate.^16^, ^17^, ^18^ At present, noble Pt‐based metals and Ru/Ir‐based oxides are the state‐of‐the‐art catalysts for HER and OER, respectively. Nevertheless, the high cost and the scarcity seriously hampered their large‐scale commercial applications. Therefore, exploring highly active and robust bifunctional electrocatalysts based on earth‐abundant elements is of significant importance to reduce the cost and improve the electrocatalytic efficiency for water splitting.

Over the past few years, considerable efforts have been devoted to developing an efficient electrocatalyst with earth‐abundant materials, such as transition metal oxides, hydroxides, carbide, sulfides, and phosphides.^19^, ^20^, ^21^, ^22^, ^23^, ^24^, ^25^, ^26^, ^27^, ^28^, ^29^, ^30^ Among them, transition metal oxides, especially for the Co_3_O_4_ material, are emerging as the one of most probable alternatives to precious metal‐based catalysts due to their natural abundance, unique 3D electronic configurations and high durability.^31^ Unfortunately, the easy self‐agglomeration and the intrinsically low electrical conductivity of purely bulk Co_3_O_4_ usually decrease the active site exposure and hinder the transport of electrons/protons during the HER or OER process, yielding unsatisfied activity.^32^, ^33^

It is widely accepted that the electrocatalytic activity of catalyst is strongly dependent on its electrochemically active surface areas and electronic states.^34^ In this regard, several strategies have been commonly utilized to optimize the catalytic activity of Co_3_O_4_. The first is to enlarge active surface area through the design of porous nanostructures in the form of nanoparticles, nanowires/nanotubes, nanomeshes, and complex architectures.^35^, ^36^, ^37^, ^38^, ^39^ In particular, 2D porous nanosheets not only possess a high percentage of coordination‐unsaturated surface atoms but also allow an efficient surface‐to‐surface contact with the electrode, ensuring more reaction sites for catalytic reaction and rapid charge transport.^17^, ^23^, ^40^ For example, Xu et al. synthesized porous cobalt oxide nanoplates through a ligand‐assisted polyol reduction process and found that they manifested OER overpotential 306 mV at 10 mA cm^−2^.^41^ The second is to hybrid Co_3_O_4_ with conductive carbon materials.^42^ Within such hybrids, not only the charge transport capability and structural stability of the whole system are improved, but also the strong coupling between active phase and carbon evokes electronic structure reconfiguration around cobalt centers, leading to enhanced catalytic activity. For example, Leng et al. reported hybrid composites consisting of Co_3_O_4_ nanoparticles uniformly grown on reduced graphene oxide, which afford a significant enhancement of OER activity due to the formation of Co—O—C bond.^43^ The third is to modulate the electronic state by heteroatom doping and defect/interface tuning.^43^, ^44^, ^45^, ^46^, ^47^, ^48^, ^49^, ^50^, ^51^, ^52^, ^53^, ^54^, ^55^, ^56^ For example, Zhang et al. introduced abundant Co vacancies into Co_3_O_4_ and demonstrate that the existence of vacancies led to electronic delocalization, providing more active catalytic sites with enhanced carrier transport.^44^ Xiao et al. filled the oxygen vacancies of Co_3_O_4_ with P by a plasma treatment and confirmed that P‐filled Co_3_O_4_ required only overpotentials of 120 mV for HER and 280 mV for OER to deliver a current density of 10 mA cm^−2^.^45^ Despite continuous achievements have been made on, the development of a robust Co_3_O_4_‐based electrocatalyst with higher catalytic activity through a delicate design toward both HER and OER still remains a significant challenge.

In the present work, for the first time, we have integrated all the above individual strategies together and developed a novel 2D N‐doped Co_3_O_4_@C nanomeshes with rich oxygen vacancies directly grown on nickel foam (N‐Co_3_O_4_@C@NF) by a simple phase‐transited lysozyme (PTL)‐driven process, representing as an exceptional active bifunctional electrocatalyst. Benefiting from the collaborative merits of highly exposed surface areas, enhanced electrical conductivity, fast charge transport kinetics, and strong synergetic effects, the self‐supporting N‐Co_3_O_4_@C@NF achieved an extremely small overpotential of 42 and 96 mV at current density of 10 mA cm^−2^ for HER and OER, respectively, which are the best catalytic performance among reported Co‐based bifunctional electrocatalyst to date. Furthermore, it enables an alkaline electrolyzer with a current density of 10 mA cm^−2^ at a very low cell voltage of 1.40 V, also a new record for the overall water splitting.

The synthesis process of N‐Co_3_O_4_@C@NF is illustrated in **Figure** **1** (see the Experimental Section in the Supporting Information for details). In the first step, PTL film was formed and strongly adheres to Ni foam (PTL@NF) substrate by mixing the stock buffer of lysozyme with Tris(2‐carboxyethyl) phosphine hydrochloride (TCEP) at room temperature. After that, PTL@NF was immersed in the cobalt nitrite solution for 12 h followed by soaking in the solution of cobalt nitrite and urea through a biomimetic mineralization process to achieve the nucleation and growth of 2D Co_3_O_4_ nanosheets on PTL@NF (Co_3_O_4_@PTL@NF). Noted that PTL not only provides abundant carboxyl and hydroxyl groups to strongly bonding to cobalt ions but also facilitated the formation of 2D nanocrystals.^48^ Finally, after a carbonization process in N_2_ at 320 °C, the oxygen‐vacancy defects can be generated on porous Co_3_O_4_ while PTL can be converted into C, resulting in the final Co_3_O_4_@C@NF.

**Figure 1:**
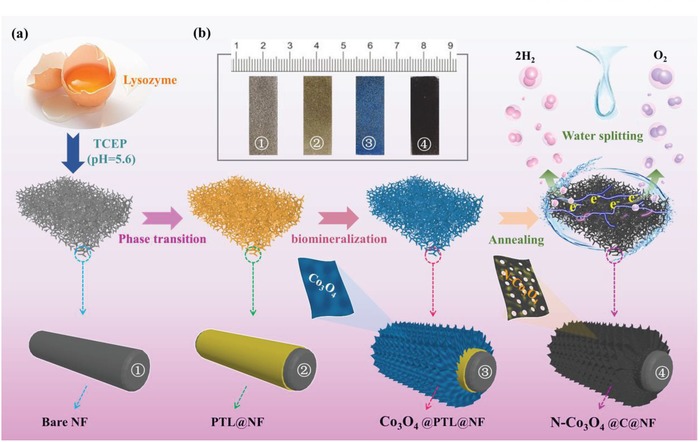
a) Schematic illustration of the synthesis procedure for N‐Co_3_O_4_@C@NF; b) photographs of bare NF, PTL@NF, Co_3_O_4_@PTL@NF, and N‐Co_3_O_4_@C@NF.

The phase and structural information of the as‐obtained samples were first investigated by X‐ray diffraction (XRD). As shown in **Figure** **2**a, N‐Co_3_O_4_@C@NF exhibit similar diffraction peaks to Co_3_O_4_@PTL@NF precursor at 31.1°, 36.8°, 59.2°, and 65.1°, corresponding to the (220), (311), (511), and (440) lattice planes of cubic spinel Co_3_O_4_ (JCPDS 01‐078‐1969), suggesting that Co_3_O_4_@PTL@NF precursor was successfully prepared by a PTL‐driven biomineralization process and further converted into N‐Co_3_O_4_@C@NF. Besides additional peaks corresponding to nickel foam, no obvious diffraction peak related to carbon appears in N‐Co_3_O_4_@C@NF products, indicating that the carbon may be amorphous. To further prove the existence of carbon, Raman spectra of the products were recorded. Noted that the products were scratched from NF for Raman testing to avoid the effect of Ni foam. Figure 2b shows the comparative Raman spectra of N‐Co_3_O_4_@C and PTL‐derived C film (N‐C) without using cobalt nitrite, in which four distinct peaks in the range of 150–800 cm^−1^ can be ascribed to the E_g_, F_2g_^1^, F_2g_^2^, and A_g_^1^ vibration modes of cubic Co_3_O_4_, while another two peaks at 1325 and 1584 cm^−1^ can be attributed to the D‐ and G‐bands of carbonaceous materials_._ 49, 50, 51 A peak around 686 cm^−1^ in N‐Co_3_O_4_@C obviously shifts toward lower wavenumber and become much broader compared to that of Co_3_O_4_@PTL, indicating that the phonon confinement effect of N‐Co_3_O_4_@C was enhanced with the Co_3_O_4_ crystal evolving from flat nanosheet to nanomesh by annealing process (Figure S1, Supporting Information).^40^ Moreover, G‐band peak in N‐Co_3_O_4_@C shifts slightly toward higher wavenumber compared to PTL‐derived C film, suggesting the strong coupling as well as the charge transfer between carbon and Co_3_O_4_ nanocrystals.^43^, ^52^, ^53^ The *I*_D_/*I*_G_ ratios of N‐Co_3_O_4_@C and PTL‐derived C film are 1.32 and 1.26, respectively, indicating that more defects contained in the N‐Co_3_O_4_@C products.^54^ The carbon content in the N‐Co_3_O_4_@C composites is determined to be 10.96% based on thermal gravimetric analysis (TGA) (Figure S2, Supporting Information).

**Figure 2:**
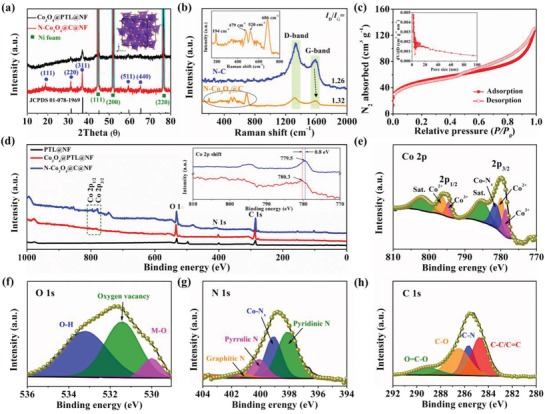
a) XRD patterns of Co_3_O_4_@PTL@NF precursor and N‐Co_3_O_4_@C@NF (Inset: crystal structure model of Co_3_O_4_); b) Raman spectra of N‐Co_3_O_4_@C and N‐C; c) the N_2_ sorption isotherm of N‐Co_3_O_4_@C@NF (Inset: the pore size distribution); d) XPS survey spectra of PTL@NF, Co_3_O_4_@PTL@NF precursor, and N‐Co_3_O_4_@C@NF, with the inset showing the shift of Co 2p; e–h) high‐resolution XPS spectra of Co 2p, O 1s, N 1s, and C1s, respectively.

Nitrogen adsorption–desorption isotherms were applied to investigate the specific surface area as well as the pore size distribution of N‐Co_3_O_4_@C composites. As shown in Figure 2c, the composites exhibit a large Brunauer–Emmett–Teller (BET) specific surface area of 195.5 m^2^ g^−1^. Correspondingly, Barrett–Joyner–Halenda (BJH) pore size distribution confirms the presence of both micropores and mesopores with the main pore diameter around 5 nm. The high porosity and a large BET surface area of the composite not only provide more abundant catalytic active sites but also are beneficial for the rapid mass/charge transportation during the electrochemical reactions.

To further confirm the chemical composition and the valence state, N‐Co_3_O_4_@C@NF was also studied by X‐ray photoelectron spectroscopy (XPS), together with the Co_3_O_4_@PTL@NF and PTL@NF. As shown in Figure 2d, the survey XPS spectra of Co_3_O_4_@PTL@NF displayed C 1s, N 1s, O 1s, and Co 2p peaks, indicating the formation of Co_3_O_4_ through a biomineralization process. Compared with that of Co_3_O_4_@PTL@NF, the position of cobalt element (Co 2p_1/2_ and Co 2p_3/2_) for N‐Co_3_O_4_@C@NF shifts to lower binding energy, implying the enhanced electron densities concentrated around the cobalt atoms possibly caused by the structural distortion via an annealing process.^40^ Figure 2e shows the high‐resolution XPS spectra of Co 2p from N‐Co_3_O_4_@C@NF sample, in which two core‐level peaks located at around 795.7 and 780.0 eV are attributed to Co 2p_1/2_ and Co 2p_3/2_, respectively. After deconvolution, the peaks around at 779.5 and 780.6 eV correspond to Co^3+^ 2p_3/2_ and Co^2+^ 2p_3/2_, respectively, while a peak around at 781.62 eV can be assigned to Co—N*_x_*.^55^ The high‐resolution XPS spectrum of O 1s in Figure 2f can be divided into three main characteristic peaks at 529.8, 531.2, and 533.2 eV, respectively, which could be attributed to the lattice oxygen from Co_3_O_4_, oxygen atoms in the vicinity of oxygen defects and the chemisorbed water, respectively.^56^, ^57^, ^58^ This result indicates that there are abundant O vacancies in the N‐Co_3_O_4_@C@NF hybrid after an annealing treatment. The high‐resolution spectrum of N 1s was shown in Figure 2g. In addition to the existence of peaks associated with pyrrolic‐N, pyridinic‐N, and graphitic‐N, a distinct peak located at 399.4 eV can be ascribed to the Co—N*_x_* feature bonds,^59^ further confirming that the N elements have been successfully doped into the Co_3_O_4_@C@NF. The pyrrolic‐N can function as metal‐coordination sites due to their lone‐pair electrons while pyridinic‐N is believed to favor in capturing of O_2_ molecules and reducing to OER overpotential.^33^ Furthermore, the C 1s spectrum was also investigated in detail (Figure 2h), which can be fitted to four peaks at 284.9, 285.6, 286.8, and 289.1 eV corresponding to sp_2_‐hybridized C=C, C—N, C—O, and C=O, respectively.

Morphology and microstructure of products were further characterized by field emission scanning electron microscopy (FESEM), atomic force microscopy (AFM), and high‐resolution transmission electron microscopy (HRTEM). As shown in Figure S3a,b (Supporting Information), a large number of Co_3_O_4_@PTL nanosheets grew uniformly and densely on the surface Ni foam. The magnified FESEM image in Figure S3c (Supporting Information) reveals that these nanosheets have a relatively smooth surface and exhibit ultrathin thickness around 12 nm, which is also verified by the observations from TEM and AFM images in Figure S3d,f (Supporting Information). A HRTEM image of Co_3_O_4_@PTL nanosheet is shown in Figure S3e (Supporting Information), in which a distinct lattice fringe spacing of 0.24 nm corresponds to the (311) plane of Co_3_O_4_. After an annealing treatment, the obtained N‐Co_3_O_4_@C products still inherit the sheet‐like morphology and keep the uniform distribution on Ni foam (**Figure** **3**a). Nevertheless, the surface of nanosheets becomes rough, indicating the formation of porous during this treatment (Figure 3b). The average thickness of the Co_3_O_4_@C nanosheets measured by AFM is about 16 nm (Figure 3c), slightly larger than that of Co_3_O_4_@PTL nanosheets, which may be due to the thermal‐induced expansion in the annealing process.^60^ Figure 3d presents the TEM image of Co_3_O_4_@C nanosheets. It can be observed that nanosheets are composed of numerous interconnected nanoparticles, representing porous mesh‐like structure. The corresponding selected area electron diffraction (SAED) pattern (Figure 3d inset) shows multiple diffraction circles, indicating the polycrystalline features of these Co_3_O_4_ nanosheets. HRTEM image taken from the nanomesh displays the lattice fringe spacing of 0.286 nm, in good agreement with the (220) plane of Co_3_O_4_ (Figure 3e–g). Remarkably, the crystal defects such as lattice distortion and stacking faults can also be found. The presence of crystal defects is believed to not only expose more active sites but also promote the adsorption of intermediates during the electrochemical reactions. The high‐angle annular dark‐field scanning transmission electron microscopy (HAADF‐STEM) and corresponding energy‐dispersive X‐ray spectroscopy (EDS) elemental mapping images revealed the coexistence and homogeneous dispersion of Co, O, C, and N within the sheet‐like framework (Figure 3h–l). Based on the above characterization results, we denoted the products obtained by annealing Co_3_O_4_@PTL as Co_3_O_4_@C nanomeshes. The interaction between Co_3_O_4_@C and NF was also investigated by powerfully durable ultrasonication of Co_3_O_4_@C@NF. As shown in Figure S4 (Supporting Information), a large number of Co_3_O_4_@C nanomeshes were still firmly coated on the surface of NF, implying their strong covalent linkage with NF rather than physical adhesion on its surface.

**Figure 3:**
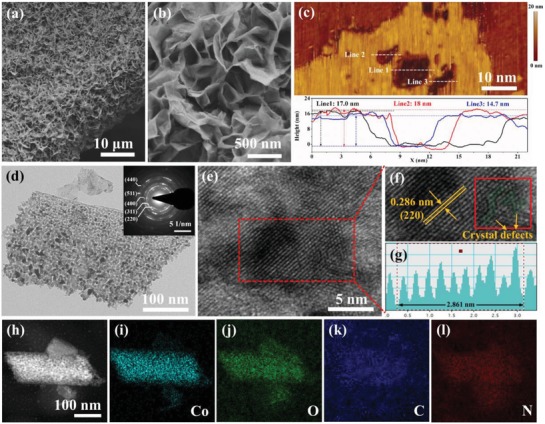
a) Low‐ and b) high‐magnified FESEM images of N‐Co_3_O_4_@C@NF; c) AFM image of N‐Co_3_O_4_@C and the corresponding line‐scan profiles; d) TEM (Inset: the corresponding SAED pattern) and e,f) HRTEM images of N‐Co_3_O_4_@C; g) the lattice spacing corresponding to the selected areas in panel (e); h–l) HAADF‐STEM image and EDS elemental mapping images of N‐Co_3_O_4_@C.

The HER electrocatalytic activities of the obtained 3D N‐Co_3_O_4_@C@NF electrode were evaluated with a three‐electrode electrochemical cell in 1.0 m KOH solution. For comparison, a series of reference samples, including the Co_3_O_4_@PTL@NF precursor, commercial 20% Pt/C deposited on Ni foam (Pt/C@NF), and PTL‐derived C film on Ni Foam (N‐C@NF) were also measured using the same method. As linear sweep voltammetry (LSV) curves shown in **Figure** **4**a, N‐Co_3_O_4_@C@NF reached the current density of 10 mA cm^−2^ at an extremely low overpotential of 42 mV versus the reversible hydrogen electrode (RHE), which is not only much lower than the 154 mV for Co_3_O_4_@PTL@NF and 174 mV for N‐C@NF but also superior to the 61 mV for commercial Pt/C@NF. Such performance also outperforms most of the state‐of‐the‐art transition metal‐based electrocatalysts (Table S1, Supporting Information). The excellent HER catalytic activity of the N‐Co_3_O_4_@C@NF is further revealed by the smallest Tafel slope of 56 mV dec^−1^, compared with 83 mV dec^−1^ for the Pt/C@NF, 121 mV dec^−1^ for the Co_3_O_4_@PTL@NF, and 221 mV dec^−1^ for the N‐C@NF (Figure 4b). To unravel more insight into the origin of the superior HER catalytic performance of N‐Co_3_O_4_@C@NF, the electrochemical surface area (ECSA) and the electrochemical impedance spectroscopy (EIS) data of the catalysts were evaluated. The ECSA was reflected from the double‐layer capacitance (*C*_dl_), which can be obtained by deriving from the cyclic voltammetry (CV) curves versus the scan rate. As revealed in Figure S5 (Supporting Information), the *C*_dl_ of N‐Co_3_O_4_@C@NF possesses the highest *C*_dl_ (92 mF cm^−2^), which is ≈8 times higher than those of other samples, suggesting more exposure of active surface sites at the solid–liquid interface. Figure 4c presents Nyquist plots of N‐Co_3_O_4_@C@NF, Co_3_O_4_@PTL@NF, Pt/C@NF, and N‐C@NF. The semicircular diameter corresponding to charge‐transfer resistance (*R*_ct_) in the EIS of N‐Co_3_O_4_@C@NF is much smaller than those of Co_3_O_4_@PTL@NF, Pt/C@NF, and N‐C@NF, meaning a favorable reaction kinetics on N‐Co_3_O_4_@C@NF electrode.

**Figure 4:**
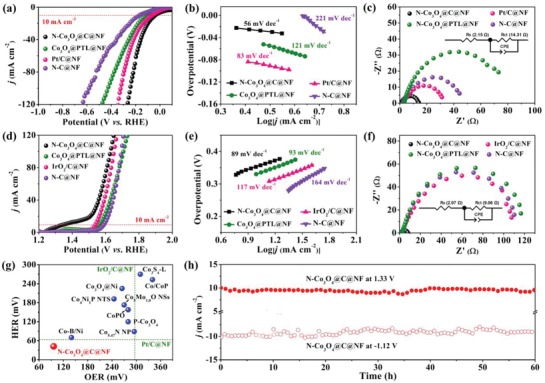
a) HER polarization curves of N‐Co_3_O_4_@C@NF, Co_3_O_4_@PTL@NF precursor, Pt/C@NF, and N‐C@NF and b) the corresponding Tafel plots, c) Nyquist plots of the N‐Co_3_O_4_@C@NF and the control electrode for HER. d) OER polarization curves and e) corresponding Tafel plots of N‐Co_3_O_4_@C@NF, Co_3_O_4_@PTL@NF precursor, Pt/C@NF, and bare N‐C@NF. f) Nyquist plots of the N‐Co_3_O_4_@C@NF and the control electrode for OER (Inset: the equivalent circuit diagram). g) Comparison of HER and OER overpotentials of N‐Co_3_O_4_@C@NF with those of reported catalysts (*j* = 10 mA cm^−2^). h) The chronoamperometry curve of N‐Co_3_O_4_@C@NF.

We then assessed the OER electrocatalytic activities of N‐Co_3_O_4_@C@NF, Co_3_O_4_@PTL@NF, N‐C@NF, and commercial IrO_2_/C deposited on NF (IrO_2_@C@NF) in 1.0 m KOH. The N‐Co_3_O_4_@C@NF catalyst still exhibits the highest electrocatalytic activity with the lowest overpotential of 96 mV to achieve a current density of 10 mA cm^−2^ compared with Co_3_O_4_@PTL@NF (332 mV), N‐C@NF (357 mV), and commercial IrO_2_/C@NF catalysts (296 mV) (Figure 4d). Table S2 (Supporting Information) gives a detailed comparison of OER performance for N‐Co_3_O_4_@C@NF and recently reported Co‐based electrocatalysts, further demonstrating its high OER activity. The corresponding Tafel plots of these catalysts also suggest the smallest Tafel value of N‐Co_3_O_4_@C@NF (89 mV dec^−1^) compared with Co_3_O_4_@PTL@NF (93 mV dec^−1^), N‐C@NF (117 mV dec^−1^), and commercial IrO_2_@C (164 mV dec^−1^), indicating a faster OER kinetic for N‐Co_3_O_4_@C@NF catalyst (Figure 4e). Such fast process could also be verified by EIS measurements (Figure 4f), from which the N‐Co_3_O_4_@C@NF possesses the smallest semicircle among all the samples, revealing its rapid charge transfer kinetics and excellent electron conductivity. In addition, a chronoamperometry measurement was performed to investigate the stability of N‐Co_3_O_4_@C@NF catalyst. As displayed in Figure 4h, the current density of 10 mA cm^−2^ was rather stable with negligible decay during the continuous HER and OER operation for 60 h, indicating its excellent durability over the long‐term electrolysis. Noted that a fluctuation during the stability test may be due to the alternate accumulation and release processes of the bubbles, which can also be observed in previously reported porous electrocatalysts.^16^

Given the excellent bifunctional HER and OER activities, a two‐electrode configuration in which N‐Co_3_O_4_@C@NF was employed as both anode and cathode was assembled to investigate the catalytic performance for overall water splitting. The catalytic performance of IrO_2_/C@NF||Pt/C@NF was also tested for comparison. It is found that the N‐Co_3_O_4_@C@NF||N‐Co_3_O_4_@C@NF couple is capable of affording a current density of 10 mA cm^−2^ at an ultralow cell voltage of only 1.40 V (**Figure** **5**a), which is not only substantially lower than that of the coupled IrO_2_/C@NF||Pt/C@NF catalyst (1.61 V) but also ranking the best as compared to other transition metal‐based bifunctional catalyst for overall alkaline water splitting as far as we know (Figure 5e and Table S3, Supporting Information). Furthermore, we have investigated Co_3_O_4_@C nanomeshes supported on nickel foam as a bifunctional electrocatalyst toward water splitting at larger current density. As shown in Figure S6 (Supporting Information), cell voltages of 1.53, 1.64, 1.77, and 1.87 V were required for N‐Co_3_O_4_@C@NF to achieve current densities of 50, 100, 200, and 500 mA cm^−2^ in overall water splitting, making it a potential candidate to be used for commercial industrial electrolyzers.^61^ Figure 5b shows an optical photograph of the overall water splitting cell, where a larger number of gas bubbles generated from the surface of both electrodes during electrolysis can be clearly observed. It should be noted that the current density around 1.3–1.4 V may be attributed to OER but not the nickel oxidation,^62^ as evidenced by faradaic efficiency measurements. As shown in Figure S7 (Supporting Information), high faradaic efficiencies of 97.4% for OER and 99.2% for HER as well as the ratio of oxygen and hydrogen close to 1:2 can be determined, indicating negligible side reactions during electrolysis. In addition, the long‐term operation performance of this N‐Co_3_O_4_@C@NF||N‐Co_3_O_4_@C@NF couple‐based electrolyzer was probed at the cell potential of 1.4 V, showing no obvious decay for a period of 24 h. The morphology of the N‐Co_3_O_4_@C@NF electrocatalyst after the durability test was also characterized. As shown in the inset of Figure 5c, the original nanosheet structure is still maintained. The chemical state of the elements in the cycled N‐Co_3_O_4_@C@NF was also investigated. As shown in Figure 5d, the deconvolution peaks of the Co 2p spectrum are centered at 779.5 and 781.3 eV, corresponding to Co^3+^ and Co^2+^, respectively. It was found that the peaks of Co^3+^ 2p_3/2_ and Co^2+^ 2p_3/2_ of the N‐Co_3_O_4_@C@NF before and after the durability test are almost the same, confirming the excellent stability of the N‐Co_3_O_4_@C@NF toward overall water splitting.

**Figure 5:**
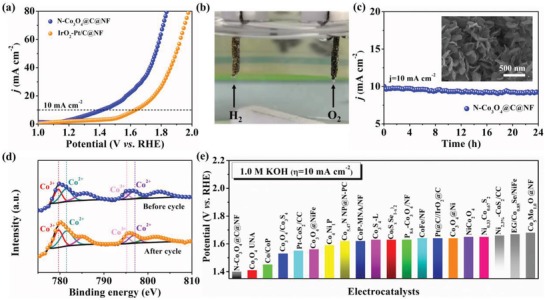
a) LSV curve of the typical two‐electrode system by employing N‐Co_3_O_4_@C@NF as both the anode and cathode in 1.0 m KOH at a scan rate of 5 mV s^−1^; commercial Pt/C@NF||IrO_2_/C@NF was also tested for comparison. b) Optical photograph showing the generation of H_2_ and O_2_ bubbles on the N‐Co_3_O_4_@C@NF from overall splitting; c) chronopotentiometric curve at a constant current density of 10 mA cm^−2^ for 24 h (Inset: FESEM image of the N‐Co_3_O_4_@C@NF after cycling test); d) high‐resolution XPS spectra of Co 2p in N‐Co_3_O_4_@C@NF before and after cycling test; e) comparison of the cell voltages to achieve a current density of 10 mA cm^−2^ for the N‐Co_3_O_4_@C@NF catalyst with other latest bifunctional catalysts.

The superior performance of the N‐Co_3_O_4_@C@NF catalyst toward overall water splitting can be related to the following reasons: (i) the incorporation of N into Co_3_O_4_ together with the coupled carbon species to form a hybrid composite could not only improve its conductivity and charge transfer capability but also generate additional defect active sites and thus ensuring intrinsically high activity;^63^, ^64^, ^65^ (ii) the porous ultrathin nanomeshes with rich oxygen vacancies and defects can effectively enlarge the accessible ECSA and favors better exposure and utilization of electrocatalytically active sites; and (iii) the intimate contact between Co_3_O_4_@C and NF as well as the self‐supported 3D open architecture without any polymeric binders kinetically facilitate the penetration of ions and mass transport and promote the release of evolved gas bubbles, which is beneficial to reaction kinetics and long‐term electrochemical stability.

In summary, we have innovatively synthesized a nitrogen‐doped Co_3_O_4_@C nanomesh with rich defects supported on nickel foam (N‐Co_3_O_4_@C@NF) via a unique PTL‐driven strategy. Due to the fact that PTL has the ability of strongly bonding to anion ions and inducing the formation of 2D nanocrystals, Co_3_O_4_@PTL nanosheets via a biomineralization process can be uniformly grown on the surface of NF, which can be further transformed into N‐Co_3_O_4_@C@NF through an annealing treatment. With their unique structural and compositional advantages, the N‐Co_3_O_4_@C@NF exhibits an exceptional activity in catalyzing both HER and OER, and thus making it possible to assemble a stable two‐electrode alkaline electrolyzer with a current density of 10 mA cm^−2^ at an ultralow cell voltage of 1.40 V. The proposed PTL‐driven strategy is convenient and can be easily extended to fabricate other porous metal oxide/C composites, which may pave a new avenue for developing highly efficient electrocatalysts toward practical water electrolysis.

## Conflict of Interest

The authors declare no conflict of interest.

## Supporting information

Supplementary
