# Supplementary material for: Phase‐Transited Lysozyme‐Driven Formation of Self‐Supported Co3O4@C Nanomeshes for Overall Water Splitting
Source: Adv Sci (Weinh). 2019 Apr 5;6(11):1900272. doi: 10.1002/advs.201900272 (PMC6548951; doi:10.1002/advs.201900272)
Supplement: Supplementary file 1 — Supplementary [file ADVS-6-1900272-s001.pdf]

Copyright WILEY-VCH Verlag GmbH & Co. KGaA, 69469 Weinheim, Germany, 2019.

# Supporting Information

for *Adv. Sci.,* DOI: 10.1002/advs.201900272

# Phase-Transited Lysozyme-Driven Formation of Self-Supported $Co_3O_4$@C Nanomeshes for Overall Water Splitting

*Yuan Ha, Lingxia Shi, Ziliang Chen, and Renbing Wu**

Copyright WILEY-VCH Verlag GmbH & Co. KGaA, 69469 Weinheim, Germany, 2019.

# Supporting Information

## Phase-Transited Lysozyme-Driven Formation of Self-Supported $Co_3O_4$@C Nanomeshes for Overall Water Splitting

*Yuan Ha, Lingxia Shi, Ziliang Chen, Renbing Wu**

**Experimental Section**

**Preparation of the Phase-Transited Lysozyme (PTL) nanofilm on the surface of Ni foam (PTL@NF).**

All the chemicals were purchased from commercial sources and directly used without further purification. In a typical synthesis, the phase transition buffer of lysozyme was freshly prepared by mixing the stock buffer of lysozyme with Tris (2-carboxyethyl) phosphine hydrochloride (TCEP) buffer.[1, 2] The cleaned Ni foam (NF) were immersed into or covered by a lysozyme phase transition buffer containing equivoluminal of 2 mg/ml lysozyme (in 10 mM of HEPES buffer, pH 7.4) and 50 mM TCEP (in 10 mM of HEPES buffer, pH 5.8). The NF substrate was then incubated at room temperature for 50 min. After that, the PTL nanofilm-coated NF was rinsed and then dried at 60 ℃ for about 30 min.

**Synthesis of N-$Co_3O_4$@C@NF via a PTL-driven strategy.**

The PTL@NF was firstly soaked in 0.1 M $Co(NO_3)_2 \cdot 6H_2O$ for 12 hours at room temperature and then taken out and dried under vacuum. After that, the PTL@NF with $Co^{2+}$ implanted was immersed in a solution containing 0.145g $Co(NO_3)_2 \cdot 6H_2O$, 0.06 g urea and 30 ml deionized water. After being stirred for 10 min, the mixture was put into a 50 ml Teflon-lined stainless steel autoclave and treated at 90 ℃ for 8 h. The formed $Co_3O_4$@PTL@NF precursor was taken out and washed thoroughly with deionized (DI) water and then dried in air at 60 ℃ for 2 hrs. Finally, the $Co_3O_4$@PTL@NF precursor was annealed at 320 ℃ in $N_2$ for 2 h with a heating rate of 2 ℃ $min^{-1}$ to obtain N-$Co_3O_4$@C@NF nanosheets. For comparison, N-C@NF was also prepared by directly annealing PTL@NF.

**Materials Characterizations**

The characterizations of the samples were performed by X-ray diffraction (s, Rigaku, Cu target, wavelength 0.154 nm), field emission scanning electron microscopy (FESEM, JEOL JSM-6700F), transmission electron microscopy (TEM, JEOL JEM-2100F). Raman scattering spectra were recorded on a LABRAM-HR Raman spectrometer excited with 532 nm $Ar^+$ laser.

X-ray photoelectron spectra (XPS) measurements were carried out using Kratos XSAM-800 spectrometer with an Mg Kα radiation source. Thermogravimetric analysis (TGA) was operated on Pyris Diamond TG/DTA system. $N_2$ sorption measurements were carried out at 77 K on Quantachrome Autosorb Auto-mated Gas Sorption System.

**Electrochemical measurements**

All measurements were carried out in a three-electrode cell with an electrochemical work station (AutoLab PGSTAT302N) at 25 ℃ in the $N_2$ or $O_2$-saturated 1.0 M KOH solution. A carbon rod and a saturated calomel electrode (SCE) were used as the counter and the reference electrode, respectively. The 3D N-$Co_3O_4$@C@NF was used as the working electrode directly. For comparison, the electrocatalytic performance of N-C@NF, commercial Pt/C loaded onto NF (Pt/C@NF), $IrO_2$/C loaded onto NF ($IrO_2$/C@NF) were also evaluated. Polarization curves were received by linear sweep voltammetry (LSV) at a scan rate of 5 mV $s^{-1}$. All potentials were calibrated with respect to RHE based on the following equation: $E_{vs.RHE} = E_{vs.SCE} + 0.2412 + 0.05916$ pH Electrochemical impedance spectroscopy (EIS) measurements for HER and OER were recorded in the frequency range of 100 kHz-0.01 Hz. The cycle durability was tested by the chronoamperometric response in the $N_2$ or $O_2$-saturated 1.0 M KOH solution. The Faradic efficiency (FE) of the electrodes was determined by the water drainage method. The evolved gas is directly collected and the corresponding gas volume is achieved by displacement of the water column. FE was finally determined by comparing the amount of experimentally quantified gas and that of theoretically calculated gas.

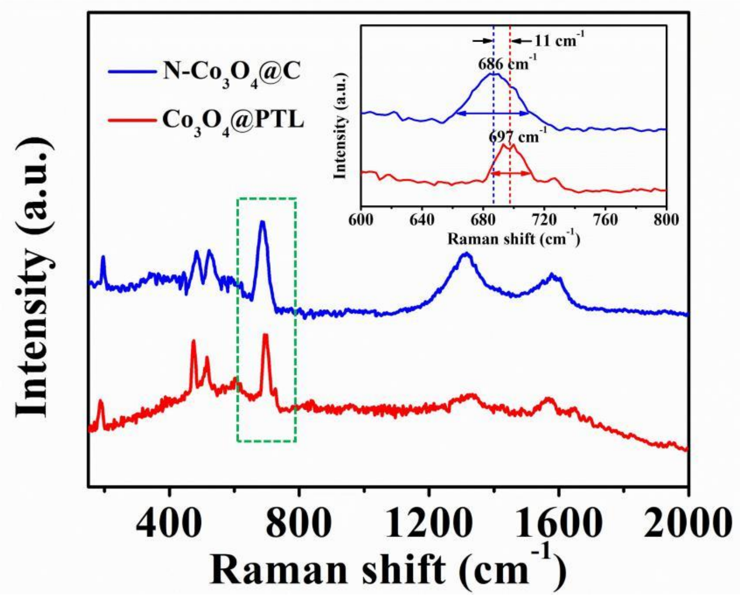


**Figure S1.** Raman spectra of N-$Co_3O_4$@C and $Co_3O_4$@PTL. The inset is the magnified view from 600 to 800 $cm^{-1}$ showing the peak broadening and peak shift for N-$Co_3O_4$@C.

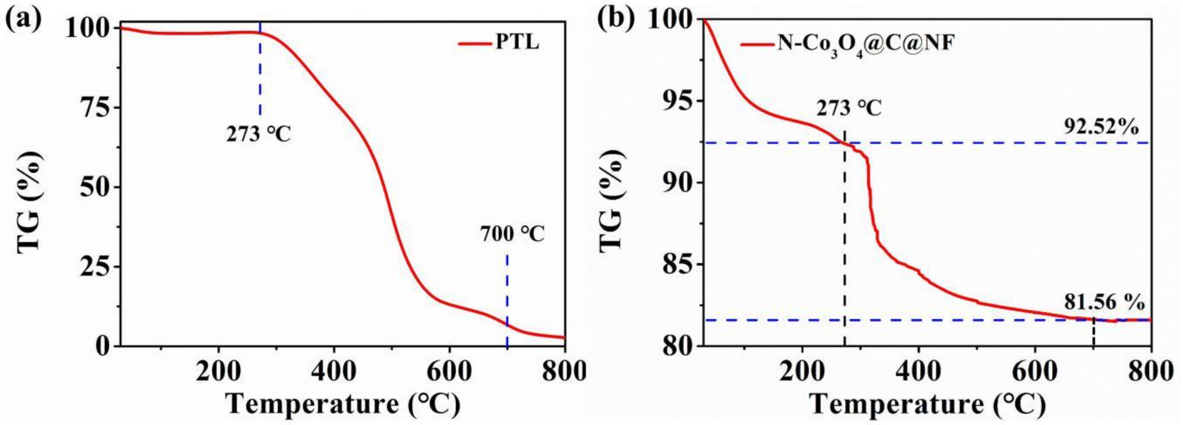


**Figure S2.** TGA curves of (a) PTL and (b) N-$Co_3O_4$@C under air atmosphere.

The carbon content in the N-$Co_3O_4$@C composites can be evaluated from the TGA results as following (Noted that the products were scratched from NF before TGA testing):

When the N-$Co_3O_4$@C composites were heated from 25 to 800 °C, there is an initial decline at 60 ℃ in TG curves, which corresponds to the mass loss of water adsorbed on the face of the materials. The TG curve of PTL shows a sharp mass decrease from 273 to 700℃, corresponding to the decomposition of PTL. As for the N-$Co_3O_4$@C@NF, a weight loss of 10.96% was found between 273 and 700℃, which could be ascribed to the combustion of PTL. After 700 ℃, the PTL nanofim was burn-out, and no distinct weight loss was observed.

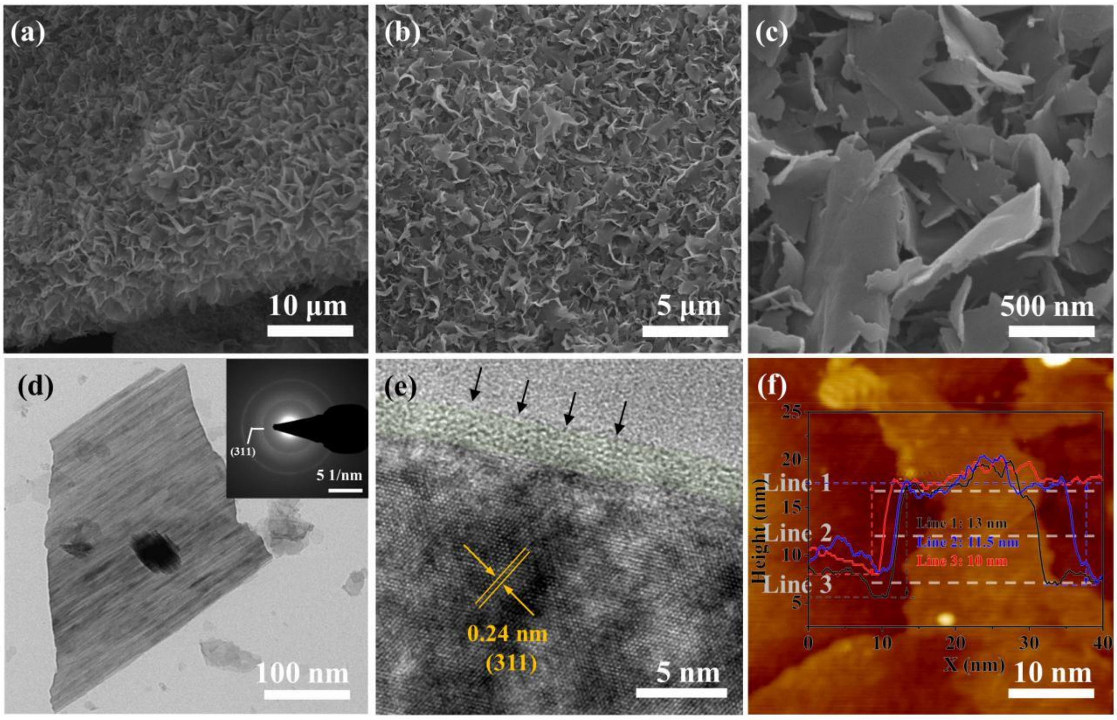


**Figure S3.** The growth of $Co_3O_4$@PTL onto the surface of NF: (a-c) FESEM images; precursor nanosheet (d) TEM (e) HRTEM and (f) AFM images $Co_3O_4$@PTL precursor. The insets in Figure S2d and Figure 2f showing selected area electron diffraction (SAED) pattern line-scan profiles, respectively.

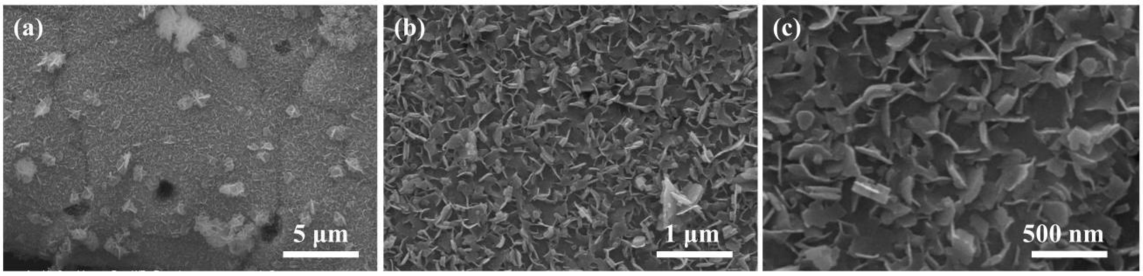


**Figure S4.** FESEM images of N-$Co_3O_4$@C@NF after after 30 min high-powered ultrasonication.

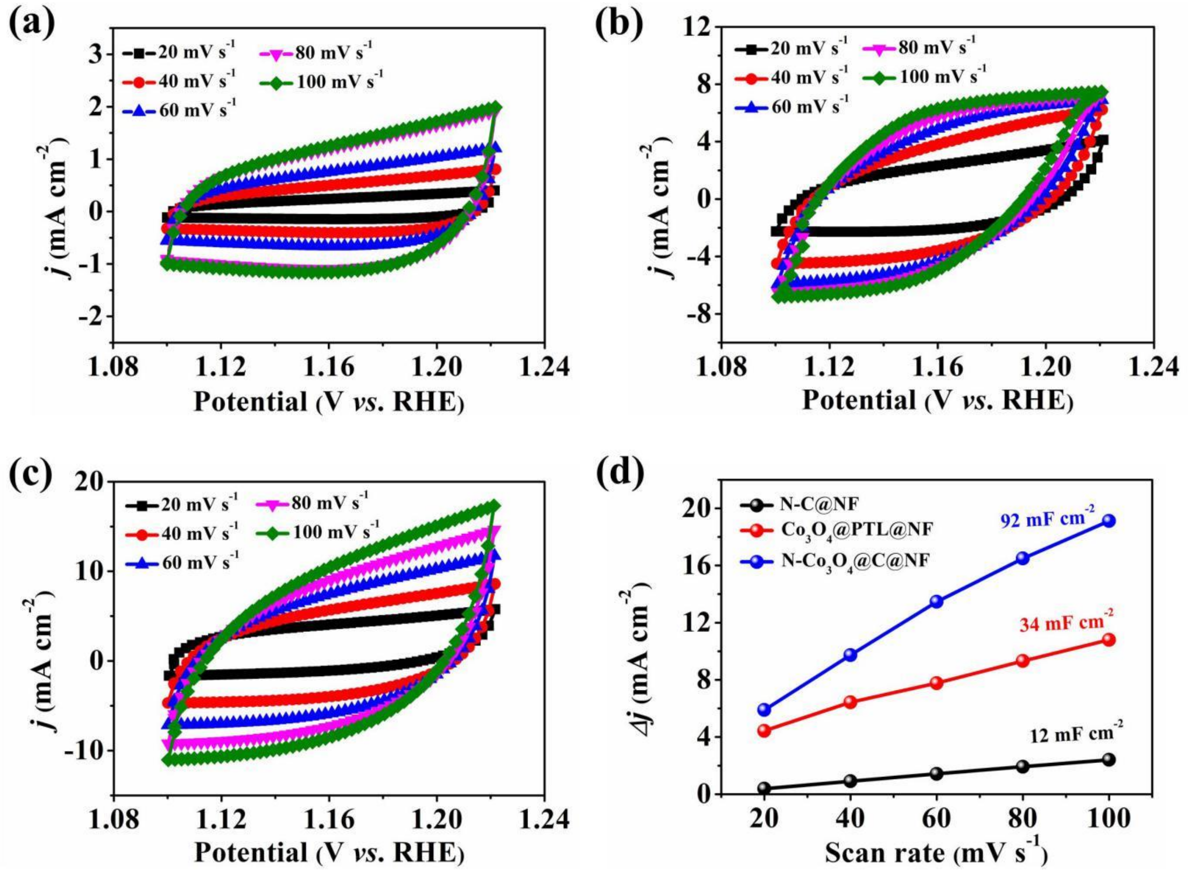


**Figure S5.** CV curves for (a) N-$Co_3O_4$@C@NF, (b) $Co_3O_4$@PTL@NF precursor and (c) N-C@NF at different scan rates: 20, 40, 60, 80, and 100 mV $s^{-1}$; and (d) corresponding plots of the capacitive currents as a function of scan rate.

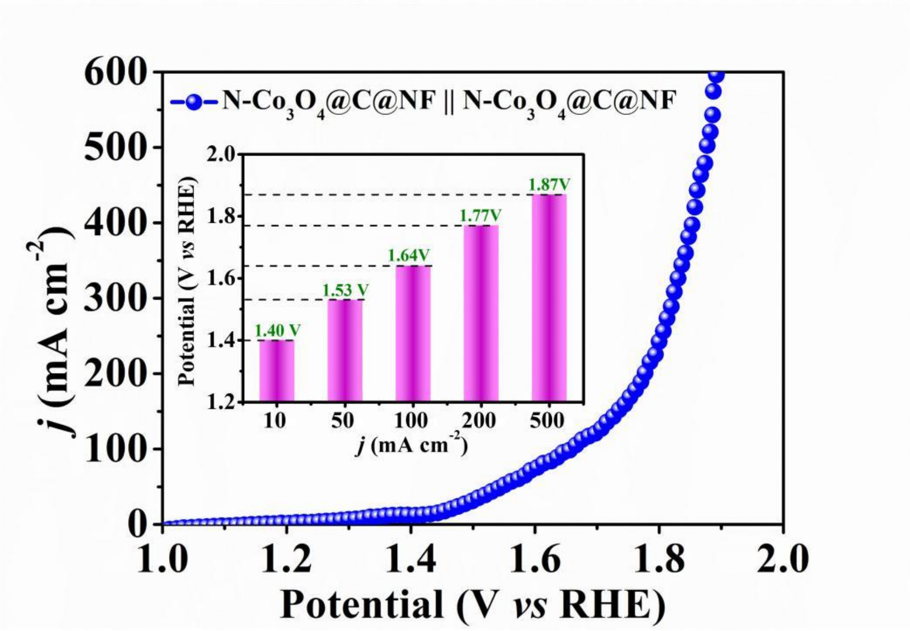


**Figure S6.** *iR*-compensated LSV curve of the typical two-electrode system by employing N-$Co_3O_4$@C@NF as both the anode and cathode in 1.0 M KOH at a scan rate of 5 mV $s^{-1}$; the inset showing the potential value at different current densities for overall splitting.

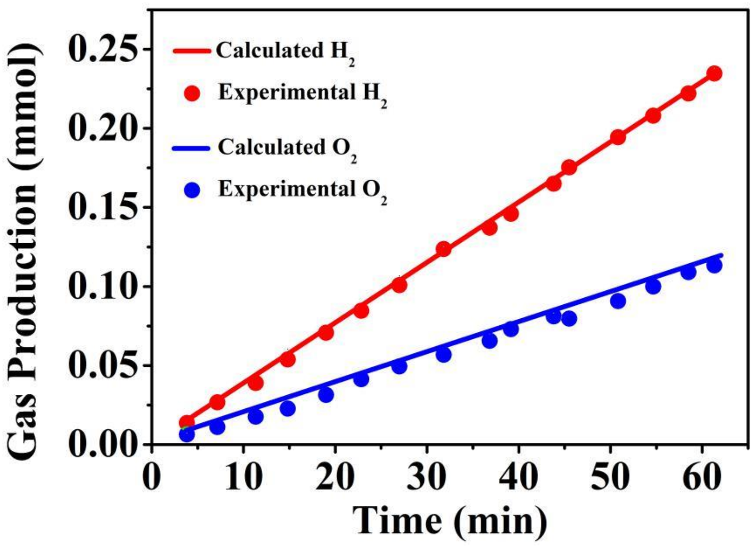


**Figure S7.** The measured and theoretical yields of $O_2$ and $H_2$ over time during electrolysis of N-$Co_3O_4$@C@NF at the current density of 10 mA $cm^{-2}$.

**Table S1.** Comparison of the electrocatalytic activity of N-$Co_3O_4$@C@NF and the reported catalysts for HER in alkaline solution.

| Catalyst | Electrolyte | Current density (*j*) | overpotential | Ref. |
|---|---|---|---|---|
| N-$Co_3O_4$@C@NF | 1 M KOH | 10 mA $cm^{-2}$ | 42 mV | This work |
| $Co_3O_4$@NiFe | 1 M KOH | 10 mA $cm^{-2}$ | 74 mV | [S3] |
| $Co_5Mo_{1.0}O$ NSs | 1 M KOH | 10 mA $cm^{-2}$ | 173 mV | [S4] |
| P-$Co_3O_4$ | 1 M KOH | 10 mA $cm^{-2}$ | 120 mV | [S5] |
| $Co_3O_4$-N-C | 1 M KOH | 10 mA $cm^{-2}$ | 139 mV | [S6] |
| $Co_4Ni_1P$ NTS | 1 M KOH | 10 mA $cm^{-2}$ | 192 mV | [S7] |
| $Co(S_xSe_{1-x})^2$ | 1 M KOH | 10 mA $cm^{-2}$ | 175 mV | [S8] |
| $CoSe_2$ | 1 M KOH | 10 mA $cm^{-2}$ | 194 mV | [S8] |
| $Co_3S_4$-L | 1 M KOH | 10 mA $cm^{-2}$ | 270 mV | [S9] |
| NiCoP/CC | 1 M KOH | 10 mA $cm^{-2}$ | 62 mV | [S10] |
| Co/CoP | 1 M KOH | 10 mA $cm^{-2}$ | 253 | [S11] |
| $CoO_x$@CN | 1 M KOH | 10 mA $cm^{-2}$ | 370 | [S12] |
| Co-NRCNTs | 1 M KOH | 10 mA $cm^{-2}$ | 62 | [S13] |
| Fe-CoP/Ti | 1 M KOH | 10 mA $cm^{-2}$ | 128 | [S14] |
| $Co_xMn_yCH$/NF | 1 M KOH | 10 mA $cm^{-2}$ | 180 | [S15] |

**Table S2.** Comparison of the electrocatalytic activity of N-$Co_3O_4$@C@NF and the reported catalysts for OER in alkaline solution.

| Catalyst | Electrolyte | Current density (*j*) | overpotential | Ref. |
|---|---|---|---|---|
| N-$Co_3O_4$@C@NF | 1 M KOH | 10 mA $cm^{-2}$ | 96 mV | This work |
| $Co_5Mo_{1.0}O$ NSs | 1 M KOH | 10 mA $cm^{-2}$ | 270 mV | [S4] |
| P-$Co_3O_4$ | 1 M KOH | 10 mA $cm^{-2}$ | 280 mV | [S5] |
| $Co_4Ni_1P$ NTS | 1 M KOH | 10 mA $cm^{-2}$ | 245 mV | [S7] |
| $Co_3O_4$ sphere | 1 M KOH | 10 mA $cm^{-2}$ | 230 mV | [S16] |
| Pt-$CoS_2$/CC | 1 M KOH | 10 mA $cm^{-2}$ | 300 mV | [S17] |
| $Co(S_xSe_{1-x})_2$ | 1 M KOH | 10 mA $cm^{-2}$ | 283 mV | [S8] |
| $CoSe_2$ | 1 M NaOH | 10 mA $cm^{-2}$ | 339 mV | [S8] |
| $Co_3S_4$-L | 1 M KOH | 10 mA $cm^{-2}$ | 310 mV | [S9] |
| Co/CoP | 1 M KOH | 10 mA $cm^{-2}$ | 340 mV | [S11] |
| NCNT/CoO-Co | 1 M KOH | 10 mA $cm^{-2}$ | 380 mV | [S18] |
| $Co_4N$/CNW/CC | 1 M KOH | 10 mA $cm^{-2}$ | 310 mV | [S19] |
| $CoO_x$-MoC | 1 M KOH | 10 mA $cm^{-2}$ | 330 mV | [S20] |
| CoP film | 1 M KOH | 10 mA $cm^{-2}$ | 345 mV | [S21] |
| $Co_3S_4$ HNSs | 1 M KOH | 10 mA $cm^{-2}$ | 307 mV | [S22] |

**Table S3.** The two-electrode system for overall water electrolysis in alkaline solution.

| Catalyst | Electrolyte | Current density (*j*) | Potential | Ref. |
|---|---|---|---|---|
| N-$Co_3O_4$@C@NF | 1 M KOH | 10 mA $cm^{-2}$ | 1.40 V | This work |
| $Co_5Mo_{1.0}O$ NSs | 1 M KOH | 10 mA $cm^{-2}$ | 1.68 V | [S4] |
| $Co_3O_4$@NiFe | 1 M KOH | 10 mA $cm^{-2}$ | 1.56 V | [S3] |
| $Co_4Ni_1P$ nanotubes | 1 M KOH | 10 mA $cm^{-2}$ | 1.59 V | [S7] |
| $Co_3O_4$ @Ni | 1 M KOH | 10 mA $cm^{-2}$ | 1.64 V | [S16] |
| Pt-$CoS_2$/CC | 1 M KOH | 10 mA $cm^{-2}$ | 1.55 V | [S17] |
| $Co(S_xSe_{1-x})_2$ | 1 M KOH | 10 mA $cm^{-2}$ | 1.63V | [S8] |
| $Co_3O_4/Co_3S_4$ | 1 M NaOH | 10 mA $cm^{-2}$ | 1.53 V | [S23] |
| $Co_3S_4$-L | 1 M KOH | 10 mA $cm^{-2}$ | 1.63 V | [S9] |
| Co/CoP | 1 M KOH | 10 mA $cm^{-2}$ | 1.45 V | [S11] |
| $NiCo_2O_4$ | 1 M KOH | 10 mA $cm^{-2}$ | 1.65 V | [S24] |
| EG/$Co_{0.85}$Se/NiFe | 1 M KOH | 10 mA $cm^{-2}$ | 1.67 V | [S25] |
| $P_{8.6}$-$Co_3O_4$/NF | 1 M KOH | 10 mA $cm^{-2}$ | 1.63 V | [S26] |
| $Co_3O_4$ UNA | 1 M KOH | 10 mA $cm^{-2}$ | 1.41 V | [S27] |
| CoP-MNA/NF | 1 M KOH | 10 mA $cm^{-2}$ | 1.62 V | [S28] |
| $Co_{5.47}$N NP@N-PC | 1 M KOH | 10 mA $cm^{-2}$ | 1.62 V | [S29] |
| CoFe/NF | 1 M KOH | 10 mA $cm^{-2}$ | 1.64 V | [S30] |
| $Ni_{2.3\%}$-$CoS_2$/CC | 1 M KOH | 10 mA $cm^{-2}$ | 1.66 V | [S31] |
| $Ni_{0.33}Co_{0.67}S_2$ | 1 M KOH | 10 mA $cm^{-2}$ | 1.65 V | [S32] |

References

[1] D. Wang, Y. Ha, J. Gu, Q. Li, L. Zhang, P. Yang, *Adv. Mater*. **2016**, *28*, 7414-7423.

[2] Y. Ha, J. Yang, F. Tao, Q. Wu, Y. Song, H. Wang, P. Yang, *Adv. Funct. Mater.* **2018**, *28,* 1704476.

[3] S. Wang, J. Wu, J. Yin, Q. Hu, D. Geng, L. M. Liu, *ChemElectroChem* **2018**, *5,* 1357-1363.

[4] Y. Zhang, Q. Shao, S. Long, X. Huang, *Nano Energy* **2018**, *45*, 448-455.

[5] Z. Xiao, Y. Wang, Y. C. Huang, Z. Wei, C. L. Dong, S. Wang, *Energy Environ. Sci.* **2017**, *10*, 2563-2569.

[6] B. You, Y. Zhang, P. Yin, D. E. Jiang, Y. Sun, *Nano Energy* **2018**, *48,* 600-606.

[7] L. Yan, L. Cao, P. Dai, X. Gu, D. Liu, L. Li, X. Zhao, *Adv. Funct. Mater.* **2017**, *27,* 1703455.

[8] L. Fang, W. Li, Y. Guan, Y. Feng H., Zhang, S. Wang, Y. Wang, *Adv. Funct. Mater.* **2017**, *27*, 1701008.

[9] M. Zhu, Z. Zhang, H. Zhang, H. Zhang, X. Zhang, L. Zhang, S. Wang, *J. colloid interface sci.* **2018**, *509*, 522-528.

[10] C. Du, L. Yang, F. Yang, G. Cheng, W. Luo, *ACS Catal.* **2017**, *7*, 4131-4137.

[11] Z. H. Xue, H. Su, Q. Y. Yu, B. Zhang, H. H. Wang, X. H. Li, J. S. Chen, *Adv. Energy Mater.* **2017**, *7*, 1602355.

[12] H. Jin, J. Wang, D. Su, Z. Wei, Z. Pang, Y. Wang, *J. Am. Chem. Soc.* **2015**, *137*, 2688-2694.

[13] X. Zou, X. Huang, A. Goswami, R. Silva, B. R. Sathe, E. Mikmeková, T. Asefa, *Angew. Chem. Int. Ed.* **2014**, *53*, 4372-4376.

[14] C. Tang, R. Zhang, W. Lu, L. He, X. Jiang, A. M. Asiri, X. Sun, *Adv. Mater*. **2017**, *29*, 160244c1.

[15] T. Tang, W. J. Jiang, S. Niu, N. Liu, H. Luo, Y. Y. Chen, J. S. Hu, *J. Am. Chem. Soc.* **2017**, *139*, 8320-8328.

[16] R. Li, D. Zhou, J. Luo, W. Xu, J. Li, S. Li, D. Yuan, J. Power Sources **2017**, *341*, 250-256.

[17] X. Han, X. Wu, Y. Deng, J. Liu, J. Lu, C. Zhong, W. Hu, *Adv. Energy Mater.* **2018**, *8*, 1800935.

[18] X. Liu, M. Park, M. G. Kim, S. Gupta, G. Wu, J. Cho, *Angew. Chem. Int. Ed.* **2015**, *54*, 9654-9658.

[19] F. Meng, H. Zhong, D. Bao, J. Yan, X. Zhang, *J. Am. Chem. Soc.* **2016**, *138*, 10226-10231.

[20] T. Huang, Y. Chen, J. M. Lee, *Small* **2017**, *13*, 1702753.

[21] N. Jiang, B. You, M. Sheng, Y. Sun, *Angew. Chem.* **2015**, *127*, 6349-6352.

[22] X. Ma, W. Zhang, Y. Deng, C. Zhong, W. Hu, X. Han, *Nanoscale* **2018**, *10*, 4816-4824.

[23] F. Meng, H. Zhong, D. Bao, J. Yan, X. Zhang, *J. Am. Chem. Soc.* **2016**.*138*, 10226-10231.

[24] X. Gao, H. Zhang, Q. Li, X. Yu, Z. Hong, X. Zhang, Z. Lin, *Angew. Chem.* **2016**, *128*, 6398-6402.

[25] Y. Hou, M. R. Lohe, J. Zhang, S. Liu, X. Zhuang, X. Feng, *Energy Environ. Sci.* **2016**, *9*, 478-483.

[26] Z. Wang, H. Liu, R. Ge, X. Ren, J. Ren, D. Yang, X. Sun, *ACS Cata.* **2018**, *8*, 2236-2241.

[27] L. Zhang, B. Liu, N. Zhang, M. Ma, *Nano Res.* **2018**, *11*, 323-333.

[28] Y. P. Zhu, Y. P. Liu, T. Z. Ren, Z. Y. Yuan, *Adv. Funct. Mater.* **2015**, *25*, 7337-7347.

[29] Z. L. Chen, Y. Ha, Y. Liu, H. Wang, H. Y. Yang, H. B. Xu, Y. J. Li, R. B. Wu, *ACS Appl. Mater. Interface* **2018**, *10*, 7134-7144.

[30] P. Babar, A. Lokhande, H. H. Shin, B. Pawar, M. G. Gang, S. Pawar, J. H. Kim, *Small* **2018**, *14*, 1702568.

[31] W. Fang, D. Liu, Q. Lu, X. Sun, A. M. Asiri, *Electrochem. Commun*. **2016**, *63*, 60-64.

[32] Z. Peng, D. Jia, A. M. Al-Enizi, A. A. Elzatahry, G. Zheng, *Adv. Energy Mater.* **2015**, *5*, 1402031.
